# Effect of 3q oncogenes *SEC62* and *SOX2* on lymphatic metastasis and clinical outcome of head and neck squamous cell carcinomas

**DOI:** 10.18632/oncotarget.13986

**Published:** 2016-12-16

**Authors:** Florian Bochen, Hana Adisurya, Silke Wemmert, Cornelia Lerner, Markus Greiner, Richard Zimmermann, Andrea Hasenfus, Mathias Wagner, Sigrun Smola, Thorsten Pfuhl, Alessandro Bozzato, Basel Al Kadah, Bernhard Schick, Maximilian Linxweiler

**Affiliations:** ^1^ Department of Otorhinolaryngology, Head and Neck Surgery, Saarland University Medical Center, Homburg (Saar), Germany; ^2^ Institute of Medical Biochemistry and Molecular Biology, Saarland University Medical Center, Homburg (Saar), Germany; ^3^ Department of General and Surgical Pathology, Saarland University Medical Center, Homburg (Saar), Germany; ^4^ Institute of Virology, Saarland University Medical Center, Homburg (Saar), Germany

**Keywords:** head and neck cancer, 3q amplification, SEC62, SOX2, prognostic biomarkers

## Abstract

Chromosome 3q26 amplification represents a frequent alteration in head and neck squamous cell carcinomas (HNSCCs). Overexpression of 3q26 encoded genes *SEC62* and *SOX2* was detected in various cancers, including HNSCCs, indicating their potential function as oncogenes. In our study, we elucidated the function of *SEC62* and *SOX2* in HNSCC patients, with a main focus on their effect on lymphatic metastasis and patient survival. We analyzed *SEC62* and *SOX2* expression in tissue specimens from 65 HNSCC patients and 29 patients with cervical cancer of unknown primary (CUP); a higher *SEC62* and lower *SOX2* expression was observed in the lymph node metastases from HNSCC patients compared with the respective primary tumor. Lymph node metastases from CUP patients showed higher *SEC62* and lower *SOX2* expression compared with lymph node metastases from HNSCC patients. When proceeding from the N1 to the N3 stage, *SEC62* expression in the lymph node metastases showed an increase and *SOX2* expression showed a decrease. Moreover, both genes showed a highly significant relevance as prognostic biomarkers, with the worst prognosis for patients with high *SEC62* and low *SOX2* expression levels. In functional analyses, knockdown of SEC62 resulted in an inhibition of HNSCC cell migration while, conversely, *SEC62* and *SOX2* overexpression stimulated cell migration. Taken together, our study showed that the expression of the 3q oncogenes *SEC62* and *SOX2* affects lymphatic metastasis and cell migration in HNSCC and CUP patients and has a high prognostic relevance in these diseases.

## INTRODUCTION

Head and neck squamous cell carcinomas (HNSCCs) account for 5% of all human malignancies and are associated with a constantly poor prognosis for many years [1]. In nearly 50% of all HNSCC patients, lymph node metastases are found at the time of diagnosis, which markedly worsens their outcome and necessitates the intensification of therapy [2, 3]. If no primary tumor is found after the initial staging procedure is completed, the disease is classified as “cancer of unknown primary” (CUP syndrome), which represents a highly malignant disease that is accompanied by a median overall survival time of only eight months [4, 5]. Although the effects of nicotine and alcohol consumption [6, 7], as well as infection of the oral mucosa with high-risk human papillomavirus [8], on the carcinogenesis of HNSCCs have been well known for many years, it is still not completely known which molecular processes drive the malignant transformation in these diseases and which differences in tumor cell biology are responsible for the different clinical phenotypes of HNSCCs compared with CUP syndrome [4, 7, 9, 10]. To uncover candidate oncogenes and tumor suppressor genes that are involved in the carcinogenesis of HNSCCs, numerous molecular genetic analyses were performed. Thus, an amplification of the long arm of chromosome 3 (3q) was identified as a characteristic genetic alteration [11, 12], which was also found in other tumor entities, including non-small-cell lung cancer (NSCLC), cervical cancer and esophageal cancer [13–15]. Subsequently, many groups aimed to identify potential target genes encoded in the 3q region and proposed *FXR1, CLAPM1, PIK3CA, EIF4G* and *P63* as potential oncogenes without, however, being able to prove a functional correlate at the level of cancer cell biology [16–19]. Our group identified *SEC62* as a further potential 3q encoded oncogene. *SEC62* encodes a transmembrane protein of the endoplasmic reticulum the precise physiological function of which in mammals is still not known. Initial studies have suggested a role for this protein in the intracellular transport of specific proteins and in calcium homeostasis [20–22]. After analyzing the gene copy number and expression of *SEC62* in tissue samples of NSCLC patients, we observed that lung cancer tissue shows a *SEC62* amplification as well as *SEC62* overexpression at both the mRNA and protein levels. In addition, the SEC62 protein level of the cancer tissue significantly correlated with a positive lymph node status and indicated poorer overall survival. Concurrently, functional analyses on lung cancer cell lines showed a marked reduction of the migratory potential of the cells after SEC62 knock-down and stimulation of HEK293 cell migration when the *SEC62* gene was overexpressed [22, 23]. Over the past several years, the role of *SEC62* as a potential oncogene has been shown in different tumors, including hepatocellular cancer [24], prostate cancer [25, 26] and HNSCCs [27].

However, little is known about the oncogenic function of *SEC62* and how this gene is able to affect cell migration and the subsequent formation of metastases. In addition to *SEC62, SOX2* constitutes another gene of the 3q26 region that encodes a transcription factor that has an essential role in embryogenesis and the maintenance of stem cell pluripotency [28, 29]. Comparable to *SEC62, SOX2* was amplified and overexpressed in different cancers, e.g., HNSCC, esophageal cancer, cervical cancer and lung cancer [30–34]. Furthermore, *SOX2* overexpression was associated with a worse prognosis in HNSCC patients [35, 36] and small-cell lung cancer [37]. Compared with *SEC62*, *SOX2* also seems to affect the migration and metastasis of cancer cells; an analysis of *SOX2* expression in HNSCC tissue specimens showed a significant correlation with positive lymph node status, [38] and artificial *SOX2* overexpression in laryngeal cancer cells stimulated their migratory potential [39, 40]. By contrast, other studies have shown a correlation between high *SOX2* expression and negative lymph node status in HNSCC patients [41, 42], as well as a favorable prognosis in NSCLC [43], gastric cancer [44] and HNSCC patients [42]. Ultimately, the role of *SOX2* in cancer cell biology and the formation of metastases remain unclear and require further studies.

In our study, we elucidated the function of both 3q26 encoded genes, *SEC62* and *SOX2*, in HNSCCs, with a main focus on their role in migration and metastasis. Therefore, we analyzed the expression level of both genes in lymph node metastases from HNSCC and CUP syndrome patients and performed functional analyses to delineate the effect of SEC62 and SOX2 on the proliferation and migration of HNSCC cells, respectively.

## RESULTS

### Comparison of clinical characteristics and survival data between HNSCC and CUP patients

**Figure 1:**
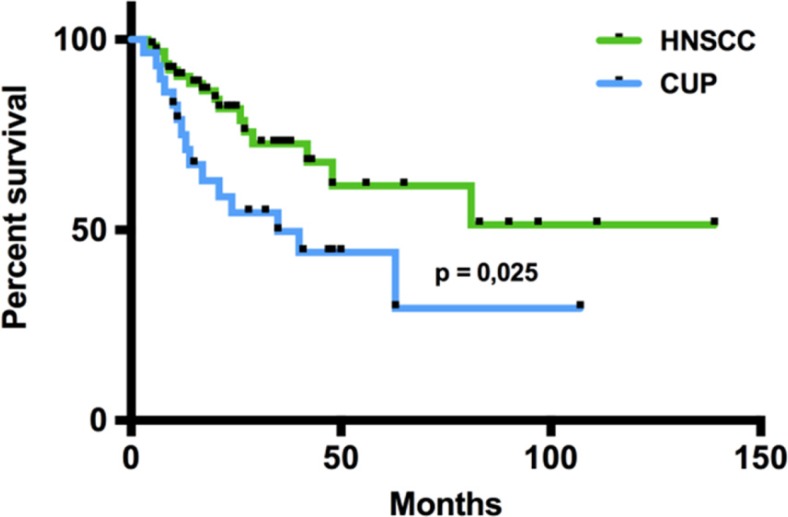
Overall survival of CUP and HNSCC patients In the Kaplan-Meier analysis, the overall survival of CUP and HNSCC patients was compared revealing a significantly worse prognosis for CUP patients (p=0,026; log-rank-test). Black dots on the survival curves represent censored data.

In total, 65 HNSCC and 29 head and neck CUP patients who were treated at the Department of Otorhinolaryngology, Head and Neck Surgery of the Saarland University Medical Center between 2004 and 2014 were included in our study. The clinical data of the patients are summarized in Table 1. CUP patients showed more advanced N stages and a higher frequency of poorly differentiated tumors, whereas the patients’ sex and median age were comparable between the HNSCC and CUP group. The majority of HNSCC patients was diagnosed at UICC stages III and IV. For both groups, surgery followed by radiation or radiochemotherapy was the treatment of choice in the majority of cases. When comparing the overall survival (OS) between the CUP and HNSCC patients, we found a significantly worse prognosis for CUP patients compared with HNSCC patients (*p* = 0.026; log-rank test, Figure 1) with median one-year survival rates of 73% (CUP patients) and 89% (HNSCC patients) and two-year survival rates of 52% (CUP patients) and 73% (HNSCC patients). Regarding the involvement of HPV, we found a higher percentage of HPV positive cases in the HNSCC group (19/65, 29%) compared to the CUP group (5/29, 17%; *p* = 0.31, Fisher's exact test) with a non-significant tendency for a survival benefit of the HPV positive patients (*p* = 0.16, log-rank test).

**Table 1:** Clinical data of HNSCC and CUP patients

		HNSCC	CUP	total
number of patients		65	29	94
Sex	male	**53** (82%)	**23** (79%)	**76** (81%)
	female	**12** (18%)	**6** (21%)	**18** (19%)
median age (years)		64,6	66,1	65,3
HPV positive		**19** (29%)	**5** (17%)	**24** (26%)
T-Stage	T1	**19** (29%)	/	/
	T2	**26** (40%)	/	/
	T3	**12** (19%)	/	/
	T4	**8** (12%)	/	/
N-Stage	N1	**14** (22%)	**5** (17%)	**19** (20%)
	N2a	**6** (9%)	**1** (3%)	**7** (8%)
	N2b	**28** (43%)	**7** (24%)	**35** (37%)
	N2c	**16** (25%)	**2** (7%)	**18** (19%)
	N3	**1** (1%)	**14** (49%)	**15** (16%)
M-Stage	M0	**64** (98%)	**29** (100%)	**93** (99%)
	M1	**1** (2%)	**0** (0%)	**1** (1%)
Grading	G2	**35** (54%)	**8** (28%)	**43** (46%)
	G3	**30** (46%)	**21** (72%)	**51** (54%)
UICCStage	0	/	/	/
	I	/	/	/
	II	**2** (3%)	/	/
	III	**28** (43%)	/	/
	IVa	**22** (34%)	/	/
	IVb	**11** (17%)	/	/
	IVc	**2** (3%)	/	/
Therapy	surgery	**8** (12%)	**4** (14%)	**12** (13%)
	surgery + RT	**23** (35%)	**14** (48%)	**37** (39%)
	surgery + RCT	**30** (46%)	**7** (24%)	**37** (39%)
	primary RT	**1** (2%)	**2** (7%)	**3** (3%)
	primary RCT	**3** (5%)	**2** (7%)	**5** (6%)

In total, 94 patients were enrolled in the study comprising 65 HNSCC and 29 CUP patients. For both groups, the patients’ gender, median age, TNM staging, grading and therapy are shown (RT – radiotherapy, RCT – radiochemotherapy).

### *SEC62* and *SOX2* expression in lymph node metastases and the primary tumors of HNSCC patients

**Figure 2:**
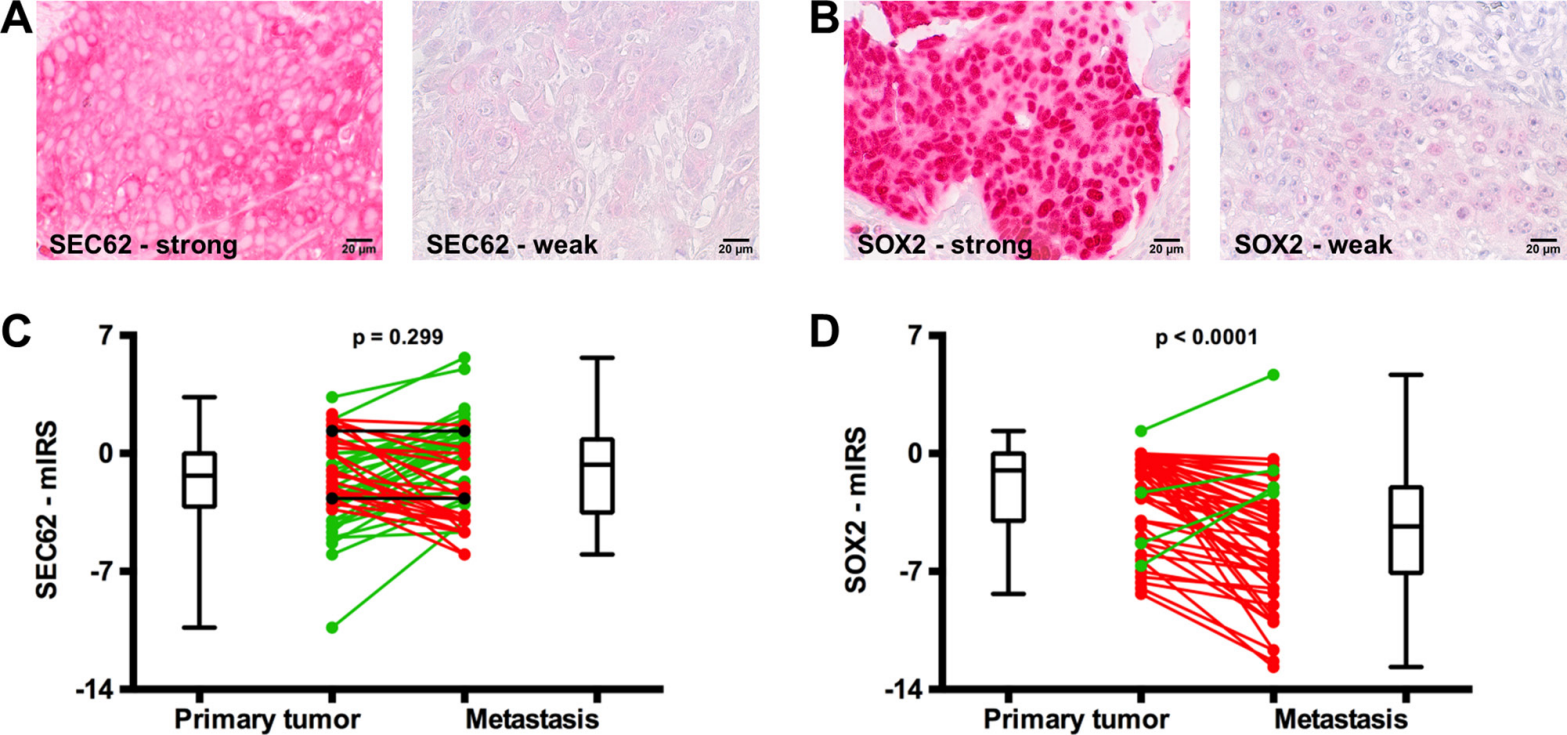
SEC62 and SOX2 expression in the primary tumor and lymph node metastases of HNSCC patients *SEC62* and *SOX2* expression was evaluated using immunohistochemistry in tissue specimens from lymph node metastases and the respective primary tumor of 65 HNSCC patients and compared with each other. (**A**) Strong (left picture) and weak expression (right picture) of *SEC62* (cytoplasmic staining signal). (**B**) Strong (left picture) and weak expression (right picture) of *SOX2* (nuclear staining signal). (**C**) SEC62-mIRS and (**D**) SOX2-mIRS for the primary tumor (left) and the metastases (right) of the HNSCC patients are shown using box and whisker blots. Each box represents the range from the first quartile to the third quartile. The median is indicated by a line. The whiskers outside the boxes represent the ranges from the minimum to the maximum value of each group. In (C) and (D), the mIRS values of the tumor and the respective metastasis are connected by a line. A green line indicates an increase in mIRS, a black line indicates an unchanged mIRS and a red line indicates a decrease in mIRS in the metastasis compared with the primary tumor.

To evaluate whether *SEC62* and *SOX2* expression exerts any influence on lymphatic metastasis of HNSCCs, we analyzed the expression levels of both genes in the primary tumor and the lymph node metastases from all 65 HNSCC patients using immunohistochemical staining. For the quantification of the staining results, we used a modified immunoreactive score (mIRS) that ranged from a minimum of -14 (weak staining) to a maximum of +14 (strong staining). Figure 2 shows examples of strong and weak SEC62 staining (Figure 2A, cytoplasmic signal) as well as strong and weak SOX2 staining (Figure 2B, nuclear signal). When comparing the SEC62- and SOX2-mIRS between the primary tumor and the metastases of the HNSCC patients, we found a weak tendency towards an elevated *SEC62* expression (60% of cases; *p* = 0.221, Mann-Whitney-test) and a significantly lower *SOX2* expression (91% of cases; *p* < 0.0001, Mann-Whitney-test) in the metastases compared with the primary tumor (Figure 2C, 2D).

### Comparison of *SEC62* and *SOX2* expression between lymph node metastases of CUP and HNSCC patients

**Figure 3:**
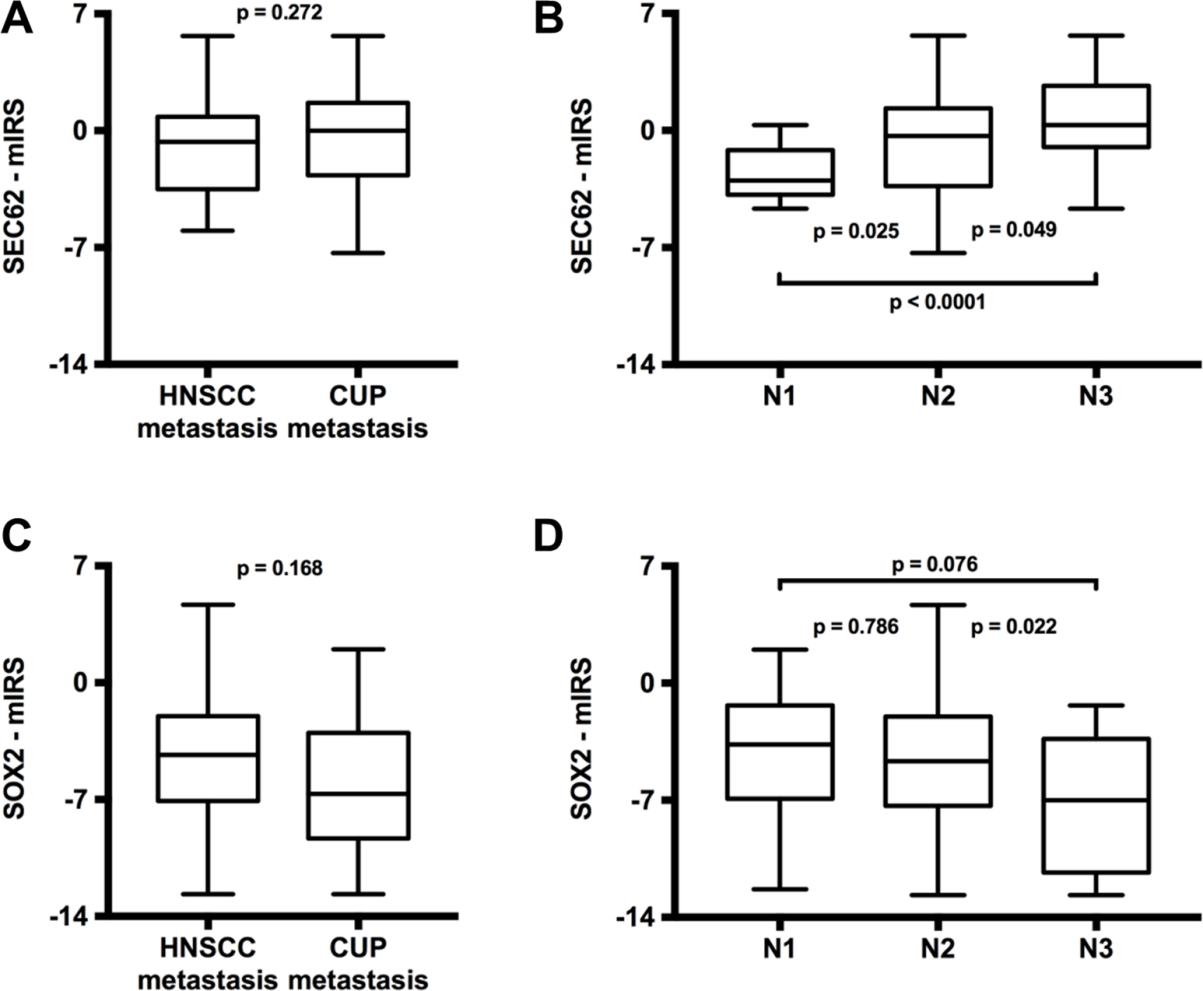
Comparison of *SEC62* and *SOX2* expression between lymph node metastases of HNSCC and CUP patients (**A** and **C**) and between different N-stages (**B** and **D**). The SEC62-mIRS (A) and SOX2-mIRS (C) for the lymph node metastases of HNSCC patients compared with the lymph node metastases of CUP patients and for all included patients grouped according to their N-stages (B and D) are shown using box and whisker blots. Each box represents the range from the first quartile to the third quartile. The median is indicated by a line. The whiskers outside the boxes represent the ranges from the minimum to the maximum value of each group.

Next, we analyzed whether the expression of *SEC62* and *SOX2* in the lymph node metastases from the HNSCC patients significantly differed from the expression of these genes in lymph node metastases from the CUP patients. Thus, we found a weak tendency towards a higher *SEC62* expression and a significantly lower *SOX2* expression in the lymph node metastases from CUP patients compared with the HNSCC patients (Figure 3A). When comparing the *SEC62* and *SOX2* expression in the lymph node metastases for all patients grouped according to the histologically proven N stages (pN1, pN2, pN3), the *SEC62* expression levels showed a stepwise increase, and the *SOX2* expression levels demonstrated a stepwise decrease when proceeding to higher N stages (Figure 3B).

### Effect of *SEC62* and *SOX2* expression on the proliferation and migration of UM-SCC1 cells

**Figure 4:**
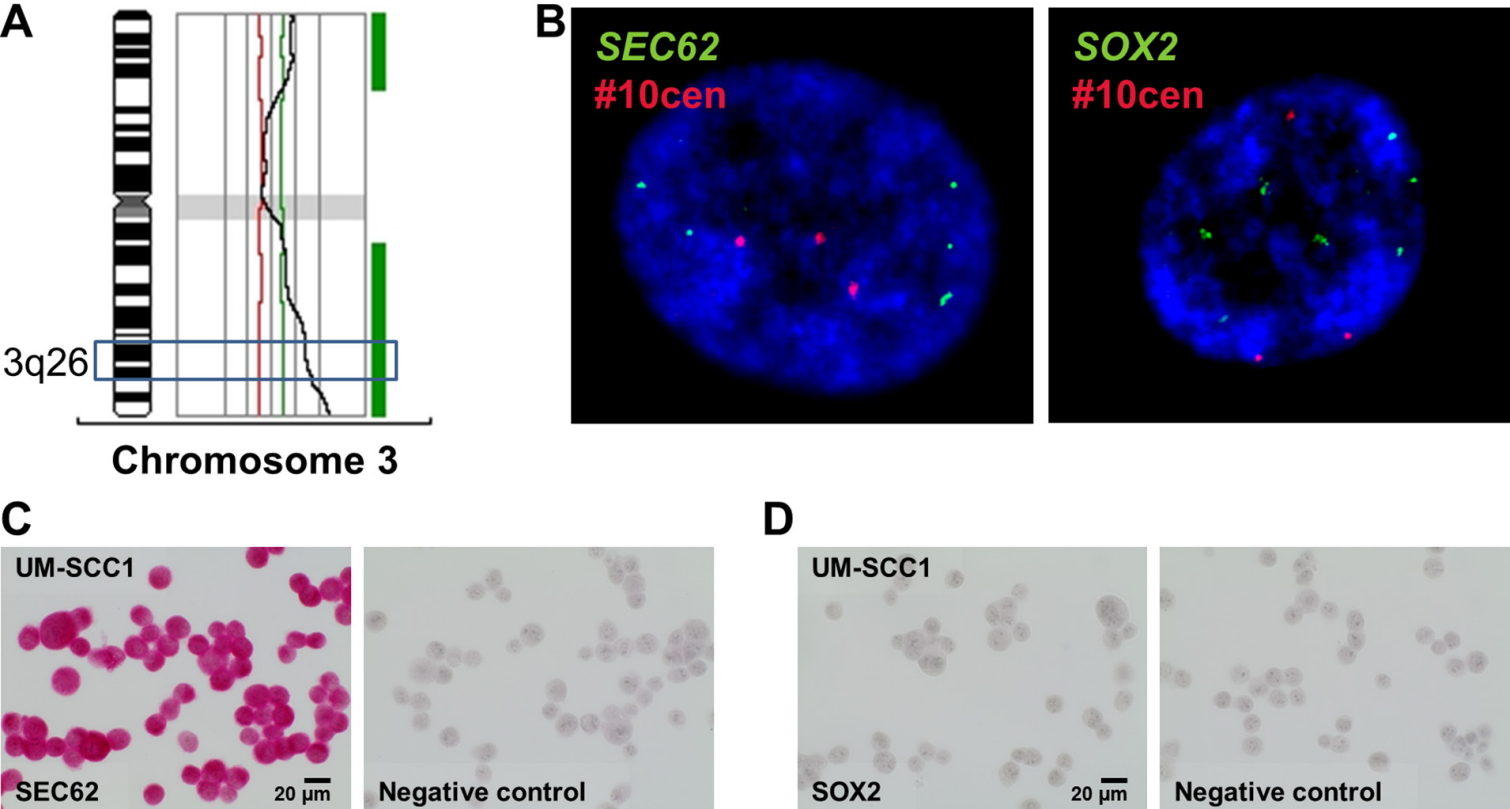
Analysis of copy number variations and expression level of SEC62 and SOX2 in UM-SCC1 cells (**A**) CGH analysis showed gains on the long arm of chromosome 3 (3q), including the 3q26 region. (**B**) FISH analysis showed amplifications of the *SEC62* gene (green signals, left picture) and the *SOX2* gene (green signals, right picture). A probe directed against the centromere region of chromosome 10 (#10cen) was used as an internal hybridization control (red signals). Nuclei (60× magnification) were counterstained with DAPI. At the protein level, we observed a strong *SEC62* expression (**C**) but no *SOX2* expression using immunocytochemistry (**D**).

**Figure 5:**
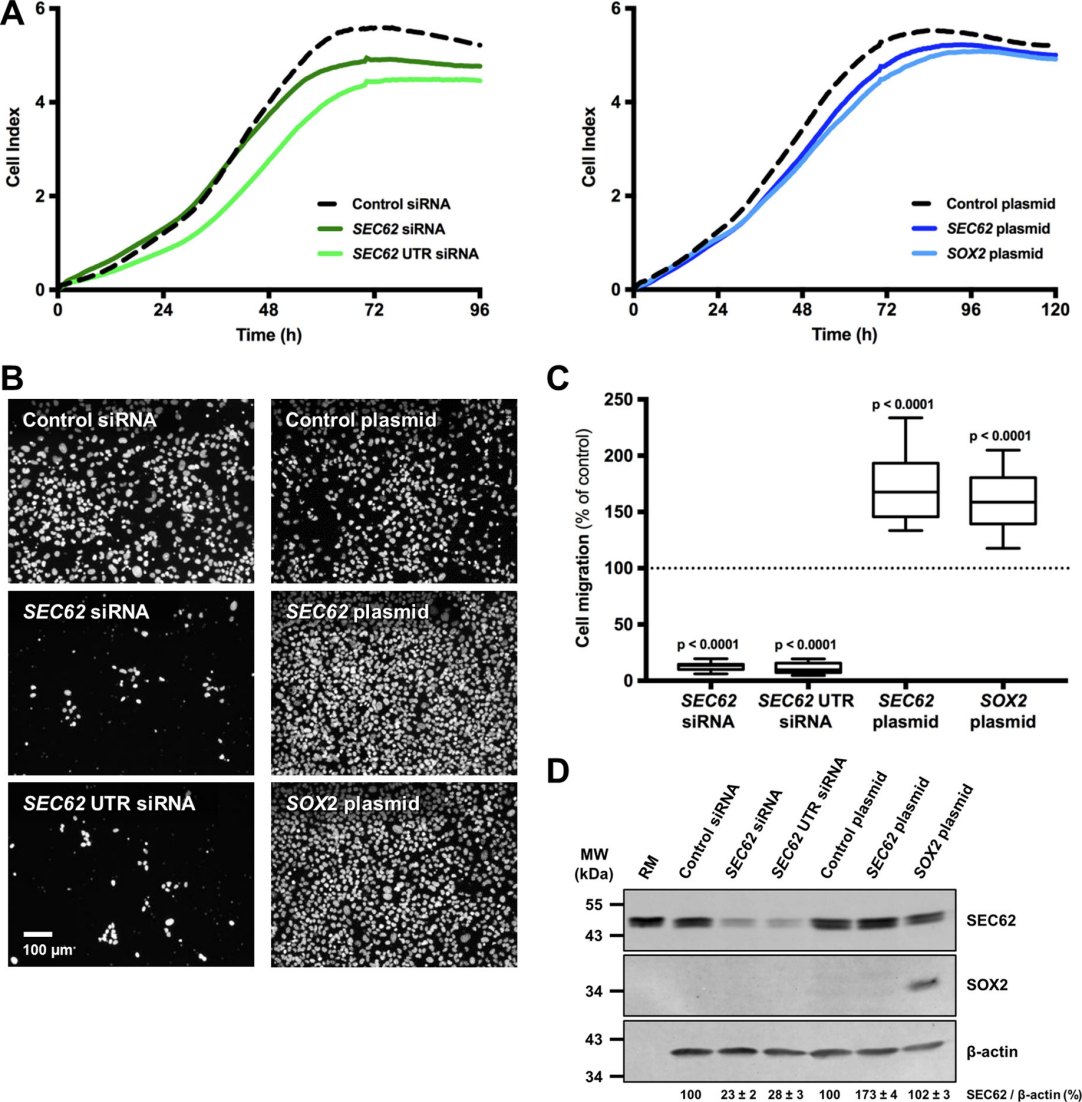
Effect of SEC62 and SOX2 expression on the proliferation and migration of UM-SCC1 cells The expression level of *SEC62* and *SOX2* was modified in UM-SCC1 cells using siRNA- and plasmid-transfection. (**A**) The cell index was measured as an indicator for cell proliferation of UM-SCC1 cells transfected with either two different *SEC62*-siRNAs, a *SEC62*-plasmid or *SOX2*-plasmid and compared with cells transfected with control siRNA or a control plasmid. (**B**) The cells that migrated through the 8-μm pores of the insert system were fixed and marked with DAPI (white dots). Representative images are shown for the transfected cells. (**C**) The number of migrated cells was quantified and is presented as a percentage of the respective control cells (= 100%) using box and whisker blots. Each box represents the range from the first quartile to the third quartile. The median is indicated by a line. The whiskers outside the boxes represent the ranges from the minimum to the maximum value of each group. (**D**) SEC62 and SOX2 protein levels of the cells were measured using western blot analyses at the end (72 h) of the migration experiments. Rough microsomes (RM) served as positive control for Sec62. The relative expression of *SEC62* per β-actin is indicated at the bottom as mean value of three identically performed experiments (*n* = 3) with the respective standard error.

To determine whether different expression levels of *SEC62* and *SOX2* affect tumor cell biology, as indicated by our immunohistochemical analyses, we performed functional analyses using UM-SCC1 cells as an *in vitro* model. First, we tested the cells for chromosomal gains and losses using CGH and analyzed both the gene copy number and the expression of *SEC62* and *SOX2* on the protein level using FISH, immunocytochemistry and western blot analyses (Figures 3, 4). Thus, we found gains on the whole chromosomal 3q region, including 3q26 (Figure 3A), amplifications of the *SEC62* and *SOX2* genes (Figure 3B) and high *SEC62* expression at the protein level (Figure 3C) without any expression of *SOX2* (Figure 3D). In karyotyping, all of the investigated UM-SCC1 cells showed a trisomy of chromosome 3. Following this characterization of UM-SCC1 cells, we reduced the SEC62 protein level in these cells using siRNA transfection and then analyzed their proliferative and migratory potential using the xCELLigence (Roche) and FluoroBlok system (BD Falcon). We found a markedly decreased migration potential of the Sec62-depleted cells without any significant change in cell proliferation compared with control siRNA transfected cells (Figure 5). By contrast, the *SEC62* overexpression induced by plasmid transfection resulted in a stimulation of cell migration compared with control plasmid-transfected cells without affecting cell proliferation. Because the UM-SCC1 cells showed no *SOX2* expression at the protein level (Figure 5D), we were restricted to analyzing the effect of *SOX2* overexpression on the migration and proliferation of the cells. We found that *SOX2* overexpression resulted in an increased migratory potential, without affecting cell proliferation (Figure 5).

### SEC62 and SOX2 are prognostic biomarkers in HNSCC and CUP patients

**Figure 6:**
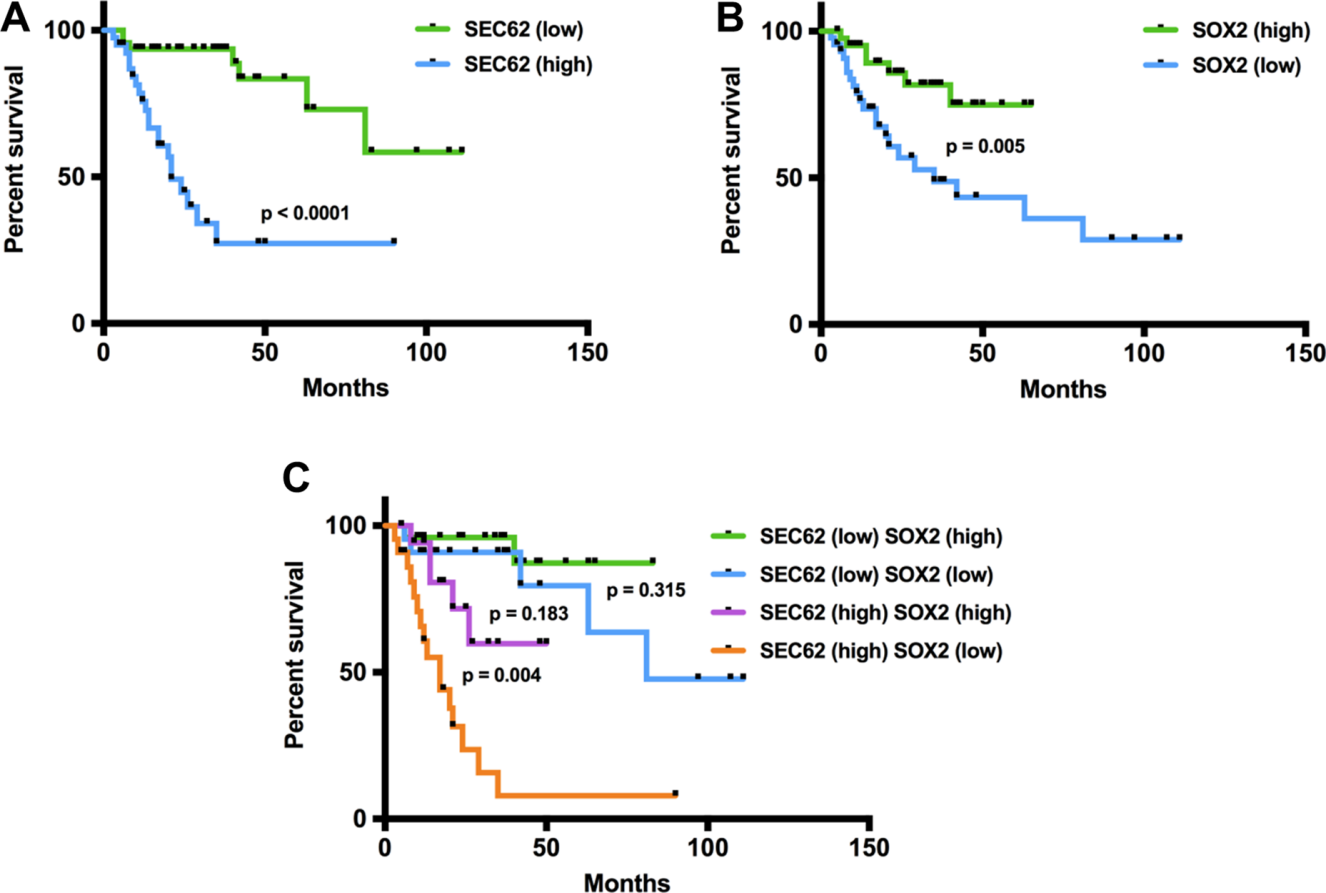
Prognostic relevance of SEC62 and SOX2 expression levels in cervical lymph node metastases of HNSCC and CUP patients In the Kaplan-Meier analysis, high *SEC62* expression and low *SOX2* expression were predictors of worse prognosis (**A**, **B**). A combination of both biomarkers showed an even higher prognostic relevance with the worst prognosis for patients who had a high *SEC62* and low *SOX2* expression level (**C**).

Following the functional analyses indicating an effect of *SEC62* and *SOX2* expression on cell migration, we correlated the results of the immunohistochemical *SEC62* and *SOX2* expression analyses in the lymph node metastases of the included HNSCC and CUP patients with their survival data (Figure 6). A high expression level of *SEC62* (SEC62-mIRS ≥ 0) was significantly correlated with a shorter overall survival (*p* < 0.0001; log-rank test), whereas high expression levels of *SOX2* (SOX2-mIRS > -5) indicated a longer overall survival (*p* = 0.002; log-rank test). When combining both markers, patients with low *SEC62* expression and high *SOX2* expression showed the best prognosis, followed by patients with low expression of both genes, patients with high expression of both genes and patients with high *SEC62* expression and low *SOX2* expression.

## DISCUSSION

3q26 amplification represents a highly frequent chromosomal alteration in HNSCCs [11, 12] and encodes the genes *SEC62* and *SOX2*, which have both been shown to be overexpressed in various cancers [13–15, 45], including HNSCCs [27, 35, 46], and to affect the metastatic potential of cancer cells [22, 23, 26, 37, 39–42, 44, 47]. Moreover, the expression levels of both genes in tumor tissue showed prognostic relevance in HNSCC patients [37, 35, 42, 48, 49]. However, the available data are limited to only a few studies with a small number of patients, particularly for *SOX2*, which showed contradictory results. In our study, we investigated the expression level of *SEC62* and *SOX2* in tissue specimens of HNSCC and CUP patients and examined their effect on the migration and proliferation of HNSCC cells *in vitro* as well as the patients’ survival data to determine their role in the process of lymphatic metastasis in HNSCC patients and their contribution to CUP biology.

When comparing the expression levels of *SEC62* and *SOX2* in the primary tumor tissue of HNSCC patients with adjacent tumor-free oral mucosa, for all cases, we found a higher expression level in tumor cells than in the healthy keratinocytes (Figure S1). These data were strongly consistent with previous studies focused on the expression of *SEC62* [27] and *SOX2* [30, 35] in healthy and cancerous tissue of the head and neck region and consistently showed an overexpression of both genes in HNSCC tissue.

For *SOX2*, immunohistochemical analyses revealed significantly lower expression in the metastases of HNSCC patients compared with the respective primary tumor (*p* < 0.0001; Figure 2D), a significantly lower expression in the metastases of CUP compared with HNSCC patients (*p* = 0.01; Figure 3A) and a gradual decrease in the expression level from N1 to N3 cervical lymph node status (Figure 3B). These data indicated the stimulation of lymphatic metastasis in HNSCC patients by low expression levels of *SOX2*. Comparable results have been published by other groups for both gastric cancer patients [44] and HNSCC patients [41, 42] in terms of a significant correlation between high *SOX2* expression levels and a negative lymph node status. However, our functional analyses on UM-SCC1 cells did not support this hypothesis; by contrast, we showed stimulation of cell migration when the *SOX2* gene was overexpressed by plasmid transfection (Figure 5). Similar effects of *SOX2* overexpression on the migration of HNSCC cells have previously been reported by other groups [37, 39, 40, 47], and an inhibitory effect of *SOX2* overexpression on HNSCC cell migration has also been published [42]. Ultimately, the precise effect of SOX2 on the migration and metastasis of HNSCC cells remains unclear because the results of the available studies, including our own study, are contradictory. Potential explanations for these discrepancies in the descriptive analyses of the patient tissue and in cell culture experiments could be the heterogeneity of the relatively small patient cohorts, the molecular diversity of the applied HNSCC cell lines or the different methods that were used for *SOX2* down- and upregulation. Finally, further studies enrolling a larger number of patients and a wider spectrum of different HNSCC cell lines are needed to uncover the role of SOX2 on the lymphatic metastasis of HNSCCs.

For *SEC62*, immunohistochemical analyses of the tissue specimens revealed a tendency towards higher expression levels in the lymph node metastases of HNSCC patients compared with their respective primary tumor (Figure 2C), a tendency towards higher expression in the lymph node metastases of CUP compared with HNSCC patients (Figure 3A) and a gradually increasing expression level from N1 to N3 cervical lymph node status (Figure 3B). Taken together, these data indicate the stimulation of lymphatic metastasis in HNSCC and CUP patients by high *SEC62* expression levels, although these correlation analyses did not reach a comparably high significance, as shown for *SOX2*. Consistent with these findings, we found stimulation of the migratory potential of UM-SCC1 cells when the *SEC62* gene was overexpressed and an inhibition of cell migration following *SEC62* silencing without any effect on cell proliferation (Figure 5). These data were strongly consistent with the findings obtained in previous studies, which reported a correlation of high *SEC62* expression levels with a positive lymph node status in NSCLC patients [23], a stimulation of the migratory potential of HEK293 cells by *SEC62* overexpression and inhibition of the migration of prostate cancer, glioblastoma, NSCLC and fibrosarcoma cells by *SEC62* silencing [26]. However, the mechanism underlying how SEC62 influences the cellular process of migration and subsequent lymphatic metastasis remains unclear. Uncovering this missing link between SEC62 and cell migration is essential for developing molecular strategies that can inhibit the migration-stimulating function of SEC62 and will be addressed in future studies.

Finally, the analysis of the patients’ survival data showed a prognostic relevance for SEC62 and SOX2 in terms of a significantly longer overall survival when the *SEC62* gene was expressed at a low level (*p* < 0.0001) and the *SOX2* gene was expressed at a high level (*p* = 0.003) in the lymph node metastases of HNSCC and CUP patients (Figure 6). For *SEC62*, these data confirmed our previously reported findings for a smaller cohort of HNSCC patients, in which we analyzed *SEC62* expression in tissue specimens of the primary tumor [27]. For *SOX2*, some studies were consistent with our results and reported a better outcome of HNSCC patients with high *SOX2* expression levels in the primary tumor [42, 48, 49]. By contrast, other studies found a worse prognosis for HNSCC patients with high *SOX2* expression in the primary tumor [35, 50]. However, these findings are only partially comparable to our results because no previous study has addressed the prognostic relevance of *SEC62* and *SOX2* expression in the lymph node metastases of HNSCC patients. Finally, the significance of *SOX2* as a prognostic biomarker in HNSCC patients remains unclear, as the currently published studies are contradictory, although the majority of these studies reported high *SOX2* expression levels as an adverse prognostic factor in this tumor [50].

Taken together, this study has shown that the expression of both *SEC62* and *SOX2* shows differences between the primary tumor and lymph node metastases of HNSCC patients and between the lymph node metastases of HNSCC and CUP patients. This may potentially contribute to the different clinical courses of these two tumors because functional analyses revealed a crucial effect of both genes on the migration of HNSCC cells. Furthermore, we found prognostic relevance for the expression level of both genes in the lymph node metastases of HNSCC and CUP patients. However, how this knowledge can be transferred to the clinical management of HNSCC patients and whether SEC62 and SOX2 can be used as therapeutic targets in this entity will be addressed in future studies.

## MATERIALS AND METHODS

### Patient characteristics and tissue samples

In total, 65 HNSCC patients and 29 CUP syndrome patients were enrolled in this study. For both groups, the patients were matched for age and gender. For further analyses, formalin-fixed paraffin-embedded (FFPE) tissue samples of cervical lymph node metastases, and for HNSCC patients, samples from the primary tumor were obtained.

For all included cases, the histological diagnosis was squamous cell carcinoma (SCC). The primary tumor localizations for the HNSCC group were tonsil (*n* = 24), tongue base (*n* = 14), hypopharynx (*n* = 12), larynx (*n* = 10), floor of the mouth (*n* = 4) and tongue border (*n* = 1). The median follow-up time for all patients was 30 months (31 months for the HNSCC group and 29 months for the CUP group). The Saarland Medical Association ethics review committee approved the scientific use of the patients’ tissue and clinical data. All experiments were performed according to relevant guidelines and regulations. Written informed consent was obtained from all patients.

### Immunohistochemistry

FFPE tissue sections were obtained and used for immunohistochemical staining of Sec62 and Sox2. After omitting the first three 10-μm sections, consecutive 4-μm sections were obtained using a Leica RM 2235 rotary microtome (Leica Microsystems, Wetzlar, Germany), transferred onto Superfrost Ultra Plus microscope slides (Menzel-Gläser, Braunschweig, Germany) and dried in an incubator at 65°C overnight. Upon deparaffinization, heat-induced epitope retrieval was performed by microwave treatment in 10 mM citrate buffer (pH 6.0) and unspecific protein binding sites were blocked by incubation in 80 ml 0.1 M Tris/HCl (pH 7.2), 3 g BSA (Sigma Aldrich, St. Louis, MO, USA) and 20 ml FCS (Sigma Aldrich, St. Louis, MO, USA) for 30 min at room temperature. Subsequently, primary antibody incubation was performed using the same antibodies as described for western blot for 1 h at room temperature. For each staining series, a specimen taken from a subcutaneously grown tumor in mice after local injection of UM-SCC1 cells (SEC62) and a human high-grad glioma (SOX2) were used as positive and negative controls by omitting the primary antibody. Visualization was performed using the REAL^™^ detection system Alkaline Phosphatase (Dako Agilent Technologies, Glostrup, Denmark), according to the manufacturer's instructions, and the slides were counterstained with hematoxylin (Dako Agilent Technologies, Glostrup, Denmark). SEC62- and SOX2-immunoreactivity was evaluated using a modified immunoreactive score (IRS) according to Remmele and Stegner [51]. Because the majority of tumors did not show complete uniform staining reactivity, we decided to modify the conventional IRS to be able to evaluate different staining intensities on a single slide. Thus, we first identified two areas of the specimen that were representative for the two major staining intensities of the whole slide. Both areas were then evaluated according to the conventional IRS with a score ranging from 0 to 12. The modified IRS (mIRS) was calculated as the sum of both single scores ranging from a minimum of 0 to a maximum of 14. Thus, the percentage of evaluated cells in total did not exceed 100%, which explains why the mIRS cannot exceed a value of 14. Finally, the mIRS for each specimen was related to the mIRS of the respective positive control by calculating the difference between both scores (mIRS_final_ = mIRS_positive control_ – mIRS _case_), resulting in the final mIRS values ranging from a minimum of -14 to a maximum of 14.

### Immunocytochemistry

For the immunocytochemical detection of SEC62 and SOX2 in UM-SCC1 cells, slides from UM-SCC1 cells suspended in 20 ml PreservCyt solution were prepared using the ThinPrep^®^-system (Hologic Deutschland GmbH, Wiesbaden, Germany) and dried for 30 min at room temperature. Following rehydration in distilled water and a three-fold wash step in phosphate buffered saline (PBS), samples were fixed in formalin for 15 minutes. Next, epitope unmasking was performed by incubation with Target Retrieval Solution (Dako GmbH, Glostrup, Denmark) at 95°C for 30 min. After cooling to room temperature and three consecutive washing steps with PBS, unspecific binding sites were blocked with 3% BSA (bovine serum albumin) in PBS and the slides were incubated with the primary antibody (1:400 dilution in 3% BSA/PBS) for 30 min at room temperature. The same antibodies as described for western blot were used. Visualization was performed using the Dako REAL^™^ Detection System Alkaline Phosphatase/RED (Dako GmbH; Glostrup, Denmark) according to the manufacturer's instructions. Finally, the slides were counterstained with hematoxylin. Each analysis included negative controls by omission of the primary antibody. Slides were imaged using the Nikon Eclipse TE2000-S inverted microscope, Nikon Digital Sight DS-5Mc camera and NIS-Elements AR software version 3.2 (Nikon; Tokyo, Japan).

### HPV testing

To determine the involvement of HPV in the carcinogenesis of the HNSCC and CUP patients, we used a combination of HPV-DNA-PCR and a simultaneous immunohistochemical detection of p16 and Ki67 as recently described [52].

First, DNA was extracted from the FFPE samples using the QIAamp DNA Blood Mini Kit (Qiagen, Hilden, Germany) according to the manufacturer`s instructions. HPV-PCR was performed with the LightCycler 2.0 instrument (Roche, Mannheim, Germany) using the GP5+/6+ primers as described by De Roda Husman et al. [53]. SYBR green as well as gel electrophoresis were used for detection. After initial denaturation at 95°C for 15 min, 45 PCR cycles followed with denaturation at 95°C for 10 s, annealing at 45°C for 5 s and elongation at 72°C for 18 s. After amplification, a melting curve was performed at temperatures between 45°C and 95°C, with temperature increasing at a rate of 0.2°K s^−1^. Tm for the HPV16-positive control was 79°C and 82°C for the HPV18-positive control. Glyceraldehyde 3-phosphate dehydrogenase (GAPDH) PCR was performed in parallel for each sample as a control as described in Ruprecht et al. [54].

Second, for the simultaneous p16-Ki67 immuno- histochemical staining the CINtec^®^ PLUS kit (Roche mtm Laboratories, Heidelberg, Germany) was applied according to the manufacturer's instructions. Each analysis included positive and negative controls.

Only tumors with a positive HPV-DNA-PCR result as well as a dual expression of p16 and Ki67 were rated as positive.

### Cell culture and transfection

For the cell culture experiments, UM-SCC1 cells derived from a squamous cell carcinoma of the floor of the mouth were cultured in DMEM (Gibco Invitrogen, Karlsruhe, Germany) containing 10% fetal bovine serum (FBS; Biochrom, Berlin, Germany) and 1% penicillin/streptomycin (PAA, Pasching, Austria) in a humidified environment with 5% CO_2_ at 37°C. The cell line was authenticated by the German Collection of Microorganisms and Cell Culture (DSMZ) using multiplex PCR of minisatellite markers, isoelectric focusing and karyotyping in march 2016.

For gene silencing, 5.2 × 10^5^ UM-SCC1 cells were seeded onto 6-cm dishes and transfected with *SEC62*-siRNA (GGCUGUGGCCAAGUAUCUUtt; Ambion, TX, USA), siRNA directed against the 3′ untranslated region of *SEC62* (CGUAAAGUGUAUUCUGUACtt; Ambion, TX, USA) or control siRNA (AllStars Neg. control siRNA; Qiagen, Hilden, Germany) using HiPerFect Transfection Reagent (Qiagen, Hilden, Germany) according to the manufacturer's instructions. After 24 h, the medium was changed, and the cells were transfected again for an additional 24 h.

For the overexpression studies, 5.2 × 10^5^ UM-SCC1 cells were seeded onto 6-cm dishes. After 24 h, the cells were transfected with either the IRES-*GFP-SEC62* plasmid (*SEC62* plasmid), the IRES-*GFP-SOX2* plasmid (*SOX2* plasmid) or the negative control IRES-*GFP* plasmid (Control plasmid) using the X-tremeGENE HP DNA Transfection Reagent (Roche Diagnostics GmbH, Mannheim, Germany) according to the manufacturer's instructions. For all plasmids, pcDNA3 served as the parent plasmid.

### Western blot analyses

2 × 10^5^ cells were lysed in a lysis buffer (aqua dest. + 10 mM NaCl/10 mM Tris (hydroxymethyl)-aminomethan/3 mM MgCl_2_/5 % NP-40) and proteins were resolved by SDS-PAGE and identified by immunoblotting. We used an affinity-purified polyclonal rabbit anti-peptide antibody directed against the C terminus of human SEC62 (self-made), a polyclonal rabbit antibody directed against the C terminus of human SOX2 (abcam pic, Cambridge, UK) and a monoclonal mouse antibody directed against the N terminus of human b-actin (Sigma-Aldrich Co., St. Louis, MO, USA). The secondary antibodies used were ECL Plex goat anti-rabbit Cy5 or anti-mouse Cy3 conjugates (GE Healthcare, Munich, Germany). Blots were imaged using the Typhoon-Trio system and Image Quant TL software 7.0 (GE Healthcare, Munich, Germany). The SEC62, SOX2 and β-actin levels were quantified and normalized against β-actin.

### Comparative genomic hybridization (CGH)

CGH was performed as previously described [27]. Briefly, DNA from UM-SCC1 cells and reference DNA from the blood of a healthy donor were obtained using standard phenol/chloroform extraction, labeled with biotin and digoxigenin by nick translation according to the manufacturer's protocol (Roche Diagnostics, Mannheim, Germany). Hybridization was performed together with COT-1 DNA (Roche Diagnostics) to normal chromosome metaphase spreads from peripheral blood lymphocytes prepared using the following standard procedures. Post-hybridization washes were performed with 50% formamide/2× standard saline citrate (SSC), 2× SSC and 0.1 × SSC at 45°C. DNA was visualized with fluorescein-isothiocyanate (FITC, Vector Laboratories, Burlingame, CA) and rhodamine (Roche Diagnostics), respectively, and counterstained with a DAPI (4, 6-diamidino-2-phenylindole) anti-fade solution (Vector Laboratories). Fluorescence images were captured using a fluorescence microscope Olympus BX 61 and image processing was performed using the ISIS digital image analysis software system (MetaSystems, Altlussheim, Germany). Average ratio profiles were determined from the analysis of 12–15 metaphases.

### Fluorescence *in situ* hybridization (FISH)

UM-SCC1 cell culture slides were fixed using methanol/acetic acid (3:1) and pretreated with RNase A and pepsin [Sigma-Aldrich, Munich, Germany]. Next, the sections were rinsed in PBS at room temperature, followed by 4% paraformaldehyde/PBS, dehydrated, and air-dried. Bacterial artificial chromosome clones (BAC) for *SEC62* (RP11-379K17) and *SOX2* (RP11-203N24) purchased from ImaGenes (Berlin, Germany) were extracted using the NucleoBond^®^ PC100 Kit (Macherey-Nagel, Dueren, Germany). BAC clones were labeled by BioPrime^®^ DNA Labeling System (Invitrogen^™^, Life Technologies, Darmstadt, Germany) and a centromeric probe for chromosome 10 (D10Z3) as an internal hybridization control was labeled with digoxigenin by nick translation (Roche Diagnostics GmbH, Mannheim, Germany) according to the manufacturer´s instructions. Dual color hybridization was performed in 50% formamide/2× SSC and COT-1 DNA (Roche Diagnostics) at 37°C overnight. Stringency washes were performed three times in 50 % formamide/2 × SSC at 42°C and two times in 2× SSC at 42°C. Immunofluorescence detection of the biotin signals was performed using Streptavidin-FITC and biotinylated anti-Streptavidin [Vector Laboratories, Burlingame, CA] for digoxigenin using anti-dig-Cy3 (Roche Diagnostics GmbH, Mannheim, Germany), and the nuclei were counterstained with a DAPI anti-fade solution (Vector Laboratories, Burlingame, CA, USA).

### Real-time cell proliferation analysis

The xCELLigence SP system (Roche Diagnostics GmbH, Mannheim, Germany) was used for the real-time analysis of cell proliferation. This system measures the changes in impedance in specific plates, with micro electrodes covering the well bottoms (E-plates, Roche Diagnostics GmbH, Mannheim, Germany). The relative changes were recorded as the Cell Index, which is a dimensionless parameter. 1 × 10^4^ UM-SCC1 cells were transfected with either siRNA or plasmids and seeded onto a 96-well e-plate according to the manufacturer's instructions. Cells transfected with siRNA were seeded 24 h after the second transfection. Cells transfected with plasmids were seeded 24 h after the plasmid transfection. Cell proliferation was monitored for 120 h, and the data were evaluated using the RTCA 2.0 software (Roche Diagnostics GmbH, Mannheim, Germany). All cell proliferation experiments were repeated three-fold (*n* = 3), and at least a triplicate of every cell population was analyzed in each experiment.

### Migration potential analysis

Cell migration was analyzed using the BD Falcon FluoroBlok system (BD, Franklin Lakes, NJ, USA) with 8-μm pore inserts for 24-well plates. 5 × 10^4^ UM-SCC1 cells transfected with either siRNA or plasmids were loaded into the inserts in normal medium containing 1% FBS. The inserts were then placed in the wells of a 24-well plate in medium with either 10% FBS as a chemoattractant for migration or without FBS (negative control). After 72 h, the cells were fixed with methanol, the nuclei were counterstained with DAPI and the number of migrated cells was analyzed using a bottom reading fluorescence microscope. Three representative images of each insert were obtained, and the number of migrated cells was quantified using NIS-Elements AR software version 3.2 (Nikon, Tokyo, Japan). All cell migration experiments were repeated three-fold (*n* = 3), and a triplicate of every cell population was analyzed in each experiment.

### Statistical analysis

Statistical analysis of the overall survival was performed using the Mantel-Cox test (log-rank test) using GraphPad Prism 6.0h and 7.0 (GraphPad Software, La Jolla, CA, USA). Analysis of the immune reactive scores was performed using the D’Agostino & Pearson normality test and a two-sided Mann-Whitney test. Analysis of cell proliferation and migration was performed using the D’Agostino & Pearson normality test and a two-sided, unpaired Student's *t*-test. *P*-values < 0.05 were considered statistically significant (α = 0.05).
